# 20-Hydroxyecdysone and Receptor Interplay in the Regulation of Hemolymph Glucose Level in Honeybee (*Apis mellifera*) Larvae

**DOI:** 10.3390/metabo13010080

**Published:** 2023-01-03

**Authors:** Jing Yu, Hongfang Wang, Wenfeng Chen, Hongyu Song, Ying Wang, Zhenguo Liu, Baohua Xu

**Affiliations:** College of Animal Science and Technology, Shandong Agricultural University, Tai’an 271000, China

**Keywords:** 20-hydroxyecdysone, metabolomics, hemolymph, glucose, ecdysone receptors

## Abstract

The hormone 20-hydroxyecdysone (20E) and the ecdysone receptors (ECR and USP) play critical roles in the growth and metabolism of insects, including honeybees. In this study, we investigated the effect of 20E on the growth and development of honeybee larvae by rearing them in vitro and found reduced food consumption and small-sized pupae with increasing levels of 20E. A liquid chromatography-tandem mass spectrometry (LC-MS/MS)-based analysis of widely targeted metabolomics was used to examine the changes in the metabolites after an exogenous 20E application to honeybee larvae and the underlying mechanisms. A total of 374 different metabolites were detected between the control group and the 20E treatment group, covering 12 subclasses. The most significant changes occurred in 7-day-old larvae, where some monosaccharides, such as D-Glucose and UDP-galactose, were significantly upregulated. In addition, some metabolic pathways, such as glycolysis/gluconeogenesis and galactose metabolism, were affected by the 20E treatment, suggesting that the 20E treatment disrupts the metabolic homeostasis of honeybee larvae hemolymph and that the response of honeybee larvae to the 20E treatment is dynamic and contains many complex pathways. Many genes involved in carbohydrate metabolism, including genes of the glycolysis and glycogen synthesis pathways, were downregulated during molting and pupation after the 20E treatment. In contrast, the expression levels of the genes related to gluconeogenesis and glycogenolysis were significantly increased, which directly or indirectly upregulated glucose levels in the hemolymph, whereas RNA interference with the 20E receptor EcR-USP had an opposite effect to that of the 20E treatment. Taken together, 20E plays a critical role in the changes in carbohydrate metabolism during metamorphosis.

## 1. Introduction

Similar to other holometabolic insects, honeybees develop in four different phases (egg, larva, pupa and adult), separated by metamorphic molts. The larval phase is mainly a period of feeding and growth. During insect development, the feeding larvae experience a significant increase in body weight, which ultimately determines the size of the future adult [1]. Ecdysone and juvenile hormones control various physiological events during an insect’s molt and metamorphosis [2,3,4,5,6]. Extensive studies suggest that endocrine signaling pathways are active in insects and regulate their nutritional homeostasis for insect growth and development [7,8,9,10,11,12,13,14,15]. The ecdysone is the main regulator of the developmental transitions in insects, and the pulsatile secretion of ecdysone drives the progression of different developmental stages [16]. The active form of ecdysone, 20-hydroxyecdysone (20E), binds to its heterodimeric receptor comprising the ecdysone receptor (EcR) and ultraspiracle (USP), and the 20E–EcR–USP complex induces the transcription of early genes [5,17]. The 20E–EcR–USP complex plays an important role in controlling the metabolic responses during pupation, directing changes in the larval, pupal and adult stages [18].

Metabolites play an important role in the interaction of organisms with their environment. The metabolomes can be used to simultaneously recognize and quantify small molecules as intermediates or endpoints [19]. Several studies have demonstrated that 20E, an insect steroid hormone, plays a key role in regulating carbohydrate metabolism during the larval–pupal transition [20]. In *Bombyx mori*, 20E induces the accumulation of glycogen in the fat body of fed and food-deprived larvae and decreases the hemolymph trehalose levels by decreasing its synthesis in food-deprived larvae [8] but shows mild trehalose action in fed larvae [21]. In addition, the 20E treatment inhibited key glycolytic enzyme activity and mRNA levels in the fat body of *B. mori* larvae, whereas the knockdown of the 20E receptor EcR-USP had a contrary effect to the 20E treatment [22]. Exogenous 20E accelerates the breakdown of trehalose to glucose, which may lead to an increase in trehalose catabolism in *Antheraea pernyi* diapause pupae [23]. In *Helicoverpa armigera*, it inhibits glycolysis and glycogen synthesis by suppressing the expression of related genes, thereby blocking the metabolic output of glucose in the hemolymph [24]. After the 20E treatment, several enzymes and cytoskeleton proteins are upregulated over time, reflecting an enhanced metabolism [1]. In the *mosquito*, elevated transcript levels of genes encoding major enzymes involved in lipid metabolism in the fat body of the post-blood meal phase are regulated by 20E [25]. Moreover, 20E induces autophagy (a metabolic process promoting amino acid mobilization) and apoptosis in fat bodies, providing substrates for glycoisomerization [26]. These results indicate that the steroid hormone 20E appears to play a critical role in regulating insect metabolism [27]. The hemolymph is the sole biofluid in honeybees, similar to the blood in vertebrates, and comprises liquid plasma in which hemolymph cells (hemocytes) are suspended [28]. Hemolymph, as a connective tissue, is responsible for transporting various molecules across the insect body and maintains direct contact with animal tissues [29,30]. The primary function of hemolymph is to transport nutrients, hormones and ions, and it also helps deal with invading pathogens, such as fungi, bacteria and other parasites [31,32]. Hemolymph is essential for the flow and transport of nutrients, ions and hormones in the honeybee and plays an important role in innate immune defense [33,34].

By using metabolomics, it is possible to study part of the metabolic components of an organism or biological system and to characterize the metabolic profile using analytical and computational methods [35]. Widely targeted metabolome (WTM) is a second-generation or new-generation targeted metabolome technology that differs from the existing metabolite detection methods. The technology platform has established the LC-MS/MS metabolite specimen database and integrated the advantages of non-targeted and targeted metabolite detection technologies, which can detect more than 2500 metabolites covering 18 categories and can achieve high throughput, high sensitivity and wide coverage of targeted metabolites detection [36]. Studies of individual pathways or enzymes are useful however miss subtle but important changes in the overall metabolism of an organism. By using widely targeted metabolomics, we can now examine many metabolites and pathways simultaneously [19].

Although many studies have demonstrated the involvement of 20E in the regulation of hemolymph metabolism in insects, such as fruit flies and mosquitoes, studies in bees are limited [18,25]. To study the metabolic responses of larvae after the 20E treatment, we used the widely targeted metabolomic approach to analyze the species and relative levels of larval hemolymph metabolites at different stages. The hemolymph metabolites of honeybee larvae were significantly altered by the 20E treatment, with most of the affected metabolic pathways being related to carbohydrate metabolism, amino acid metabolism and fatty acid metabolism. The 20E treatment and RNA interference (RNAi) experiments showed that 20E promoted a shift of glycolysis to gluconeogenesis and glycogenolysis by regulating the activity and gene expression of enzymes, ultimately increasing hemolymph glucose levels during metamorphosis. We believe that our findings will contribute to a deeper understanding of the role of 20E in maintaining metabolic homeostasis in honeybee larvae and will provide a reference for future studies on the 20E regulation of insect growth and development.

## 2. Materials and Methods

### 2.1. In Vitro Rearing of Larvae

Honeybee larvae (*Apis mellifera*; 1 day old) were collected from colonies in our experimental apiary at Shandong Agricultural University (Tai’an, China). All experiments were approved by the institutional animal ethics committees of Shandong Agricultural University. In order to acquire larvae of known ages, the queen bee was confined to a defined area on a comb containing empty cells for 12 h, with a queen excluder cage. Subsequently, the combs with newly laid eggs were moved to a separate place in the same colony in a queen excluder. Four days later, the combs containing the synchronized 2-day-old larvae (no more than 12 h old) were transferred to the laboratory. According to the indoor feeding in vitro method, 1-day-old larvae were moved to a temperature-appropriate tissue culture plate (48) with a 160 μL diet (Table 1). The plates were placed in a constant-temperature incubator (33 °C, relative humidity 55%), and the diet was changed daily during the experiment. Feeding was stopped when larvae reached critical weight (160–180 mg), and then they were transferred to cell culture plates (24) with sterile paper to prepare for pupation. From the first day of feeding, larvae and pupa deaths were recorded every day, and dead individuals were removed; at the end of the period, the number of living pupa and new bees was recorded, and the percentages of pupation and adult emergence were calculated.

### 2.2. 20E Treatment and Hemolymph Collection

In order to prepare for feeding, 20-hydroxyecdysone (20E) (Sigma-Aldrich, Waltham, MA, USA) was dissolved in 100% ethanol (50 mg/mL)—to prevent crystallization of the steroid—and diluted in a Bee Ringer containing 4 mM MgCl_2_, 5 mM CaCl_2_, 130 mM NaCl, 6 mM KCl, 10 mM HEPES, 25 mM glucose and 160 mM sucrose (pH 6.7, 500 mOsmol) to provide a final concentration of 10 mg/mL. The treatment group of larvae were fed a diet containing 20E (0.01 mg/mL). The larvae of the control group were fed a normal diet with the corresponding doses of ethanol diluted in the Bee Ringer as the solvent control.

The hemolymph of the larvae was collected by inserting a disposable glass microcapillary pipet (40 μL) into the side of the larva to avoid deep cuts and aspirate the hemolymph fluid by capillary action. More than five larvae were taken in each replication (three replications for each sample), and an average of 300–400 μL of hemolymph were collected from each sample, with three replicates in each group. N-Phenylthiourea (5–10 μm, Aladdin, Shanghai, China) was added to each sample to prevent oxidation of the hemolymph. The collected hemolymph was kept at −80 °C for further analysis. In order to determine the effects of 20E on larval development, the pupation and emergence rates of the larvae in each group were observed and recorded. The weights of the last instar larvae, white-eyed pupae and emerged bees were measured using electronic scales (accuracy: 0.1 mg) to investigate the larval growth. Fifty larvae were sampled from the control and treatment groups, respectively. These samples were stored at −80 °C for subsequent analysis.

### 2.3. Detection of the 20E Titer

The collected hemolymph was centrifuged at 12,000× *g* rpm for 5 min at 4 °C, and the supernatant was transferred into a new centrifuge tube. Then, 50 μL of the supernatants were diluted at a ratio of 1:4 (*v*/*v*), and a 50 μL mixture was used to detect 20E using an enzyme-linked immunosorbent assay (ELSA) kit according to the manufacturer’s instructions (Enzyme-linked Biotechnology, Shanghai, China). The experiments were performed with three biological replicates.

### 2.4. Metabolite Extraction

Samples were removed from the −80 °C refrigerator, thawed on ice, and vortexed for 10 s. Then, 50 μL of the sample was mixed with 300 μL of 20% acetonitrile-methanol internal standard extract. The mixture was vortexed for 3 min and centrifuged (12,000× *g* rpm, 4 °C) for 15 min. Then, 200 μL of the supernatant was transferred and left to stand at −20 °C for 30 min. Finally, the mixture was centrifuged (12,000× *g* rpm, 4 °C) for 3 min, and the supernatant was taken for analysis. The supernatant was collected for the UPLC-MS/MS analysis, as described previously, using a platform provided by Wuhan Metware Biotechnology Co., Ltd. (Wuhan, China) [37].

### 2.5. Conditions to the Analysis of UPLC-ESI-QTRAP-MS/MS

The data acquisition instrumentation system mainly comprises Ultra Performance Liquid Chromatography (UPLC) (EXionLC AD, https://sciex.com.cn/, accessed on 1 September 2021) and Tandem Mass Spectrometry (MS/MS) (QTRAP^®^, https://sciex.com.cn/, accessed on 1 September 2021). The chromatographic column was a Waters ACQUI T YUPLCHSS 13CI8 (1.8 µm, 2.1 mm*100 mm). The mobile phase was ultra-pure water (0.1% formic acid) in the A phase and pure acetonitrile (0.1% formic acid) in the B phase. The elution gradient was 0 min water/acetonitrile (95:5 *v*/*v*), 11 min, 10:90 *v*/*v*, 12.0 min, 10.90 *v*/*v*, 12 min, 95:5 *v*/*v*, 14.0 min, 95.5 *v*/*v*. The separation conditions were set as follows: flow rate 0.4 mL/min, column temperature 40 °C, injection volume 5 uL.

The mass acquisition conditions were as follows: electrospray ionization (ESI) at 500 °C, ion spray voltage (IS) 5500 V (positive), −4500 V (negative), ion source gas I (GSI) at 55 psi, gas II (GSII) at 60 psi, curtain gas (CUR) at 25 psi, and the collision-activated dissociation (CAD) parameter was set to high. In the triple quadrupole (Qtrap), each ion pair was scanned for detection according to the optimized declustering potential (DP) and collision energy (CE).

### 2.6. Data Processing and Analysis

Qualitative and quantitative analyses of metabolites were carried out using the methods of Wang et al. [38]. The Analyst 1.6.3 software was used to process the mass spectrometry data. The characteristic ions of each substance were screened by a triple quadrupole, the signal intensity (CPS) of the characteristic ions was obtained in the detector, and the peaks were integrated and corrected using MultiaQuant^TM^ 2.1 software. We corrected the mass spectrometry peaks detected for each metabolite in different samples to ensure qualitative and quantitative accuracy according to the information on metabolite retention time and peak shape. Principal component analysis (PCA) was performed using the built-in statistical prcomp function in the R software (www.r-project.org, accessed on 5 September 2021) and setting the prcomp function parameter scale = True to the unit variance (UV) scaled. The metabolite content data were processed by UV normalization, and then the hierarchical cluster analysis (HCA) was performed by the R software (http://www.r-project.org/, accessed on 5 September 2021) to analyze the accumulation pattern of the metabolites in different samples. Significantly different metabolites between groups were identified by VIP ≥ 1 and absolute Log2FC (fold change) ≥ 1. The VIP (variable importance in prediction) values obtained, based on the orthogonal partial least-squares discriminant analysis (OPLS-DA) results, also including scores and reciprocal plots, were conducted using the R package MetaboAnalystR [39]. The data were log transform (log2) and mean centering before the OPLS-DA. In order to avoid over-fitting, 200 random permutation experiments were conducted on the data. The identified differential metabolites were annotated using the Kyoto Encyclopedia of Genes and Genomes (KEGG) Compound database (http://www.kegg.jp/kegg/compound/, accessed on 6 September 2021), and then the annotated metabolites were then mapped to the KEGG Pathway database (http://www.kegg.jp/kegg/pathway.html, accessed on 6 September 2021). For a given list of metabolites, the *p*-value of the hypergeometric test was used to identify significantly enriched pathways.

### 2.7. RNA Interference (RNAi) in Larvae

RNAi (RNA interference) can be used in insects, and it specifically degrades the corresponding sequence of mRNA by double-stranded RNA (dsRNA), resulting in gene silencing at the post-transcriptional level [40]. By using the target sequence as a template, *dsRNA* was synthesized using a RiboMaxTM T7 system (Promega, Madison, WI, USA) according to the manufacturer’s instructions. The dsRNA of the green fluorescent protein gene (GFP) was also synthesized as a control (GenBank accession number U87974). For the *dsRNA* injections, 30 6-day-old larvae were injected with 10 μg of *dsRNA* into the hemocoel, with two injections 24 h apart, and the control larvae were injected with the same volume of *dsGFP*.

### 2.8. Hemolymph Glucose and Fat Body Glycogen Determination

The collected larval hemolymph (30 μL) was centrifuged at 5000× *g* rpm for 5 min, and the supernatant was extracted. According to the instructions of the glucose assay kit (A154-1-1, Njjcbio, Nanjing, China), 250 μL of the working solution was added to the supernatant (2.5 μL), incubated for 10 min at 37 °C in the darkness, and the absorbance was detected at 505 nm using a spectrophotometer (Multiskan SkyHigh, Waltham, MA, USA).

Fat bodies at different developmental stages were dissected under a microscope, rinsed with PBS, added to the extract buffer according to the instructions of a glycogen assay kit (BC 0340, Solarbio, Beijing, China), incubated in a metal bath at 100 °C for 10 min and shaken every 5 min to mix thoroughly. The other reagents were added after the tissue samples were completely cooled. The absorbance of each sample was detected at 620 nm using a spectrophotometer (Multiskan SkyHigh, Waltham, MA, USA).

### 2.9. GP, G-6-Pase, and α-GAL Activity Determination

A sample comprising one worker bee was homogenized in PBS at a ratio of 1:9 (*w*/*v*) for 180 s. The mixture was then centrifuged at 12,000× *g* rpm for 10 min at 4 °C. Then, the supernatant was transferred to a new centrifuge tube. Glycogen phosphorylase (GP), Glucose-6-phosphatase (G-6-Pase) and Alpha-galactosidase (α-GAL) activity assays were performed according to the instructions of the ELISA kit (Enzyme Linkage Biotechnology, Shanghai, China). Three biological replicates were performed for each experiment.

### 2.10. Quantitative Real-Time PCR (qRT-PCR) for mRNA

The total RNA of the samples was extracted using the TRIZOL^®^ reagent (TaKaRa, Dalian, China) according to the manufacturer’s instructions. First-strand cDNA for mRNA was synthesized separately using the *Evo M-MLV* Premix (Accurate Biotechnology, Hunan, China) according to the manufacturer’s protocols. The design and synthesis of all the qRT-PCR primers were performed by the Sangon Biotechnological Company (Shanghai, China). The qRT-PCR was performed on a 7500 Real-time PCR System (ABI, Waltham, MA, USA) by using a TransStart^®^ Tip Green qPCR SuperMix (TransGen Biotech, Beijing, China) according to the manufacturer’s instructions. All sequences of the PCR primers are listed in Table S1. Each treatment had at least three technical replicates. For normalization, the *β-actin* gene (GeneBank accession no. XM_017065464) was used as the reference gene. The relative expression levels of genes were calculated using the 2^−ΔΔCt^ method.

### 2.11. Statistical Analyses

The statistical analysis was performed using SAS software (v9.1; SAS Institute, Cary, NC, USA). All data are expressed as the mean ± standard error of the mean (SEM) of at least three biological replicates. The Student’s *t*-test (two groups) or one-way ANOVA followed by Dunnett’s post hoc test were performed for comparisons between the groups using the GraphPad Prism 8.0 software (San Diego, CA, USA). A significant difference is indicated at *p* < 0.05.

## 3. Results

### 3.1. Critical 20E Titer Blocked Larval Growth and Determined Body Size

**Figure 1:**
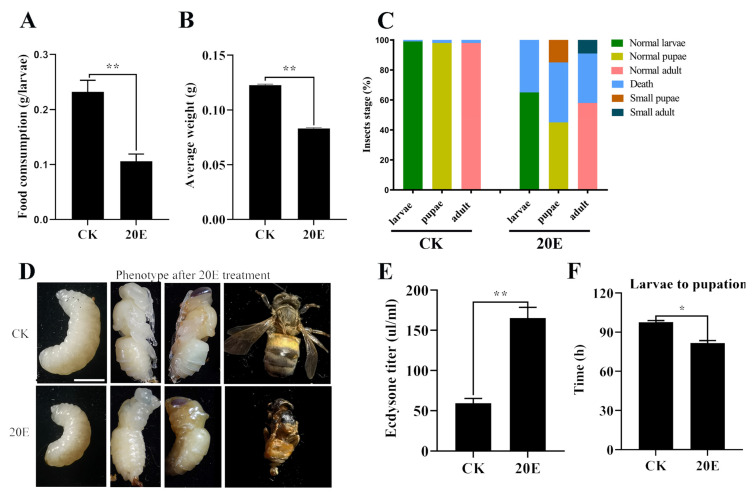
Effects of 20E treatment on food intake and growth. (**A**) Statistical analysis of the food consumption quantitated as the amount of diet eaten at the critical weight. Bee Ringer solution was used as the solvent control. CK as a control group. (**B**) Statistical analysis of average body weight of newly emerged worker bees; (**C**) percentage of distribution of different phenotypes in (**D**). Scale bar: 0.3 cm; (**E**) hemolymph ecdysone titer of 5 d, 6 d and 7 d larvae; (**F**) Statistical analysis of the pupation time from larvae to pupation. Each point indicates the mean ecdysone concentration ± SEM. The asterisks indicate significant differences from the control group (*, *p*< 0.05; **, *p* < 0.001) by using the Student’s *t*-test.

In order to investigate the function of 20E in larval metamorphosis, 2-day-old larvae were fed a diet containing 0.1% 20E until pupation. In the control group, which was fed a diet containing the same volume of solvent, the larvae developed into normal-sized pupae. The 20E-fed larvae showed a lower food intake when compared to solvent-fed larvae (Figure 1A). Compared with the control group, the body weight of the newly emerged worker bees was 35% lower in the 20E-fed larvae (Figure 1B). Furthermore, in the 20E-fed larvae, 35% of the larvae died in the last larval stage, 40% of the larvae died in the pupae stage, and 15% of the larvae developed into small-sized pupae. Only ~9% of the resultant pupae, having been fed on 20E, emerged as normal adults (Figure 1C,D). The remaining treated larvae remained as prepupae or became deformed pupae (Figure 1D). This indicated that treatment with 20E in larvae led to smaller pupae and growth inhibition, possibly due to increased 20E in the hemolymph (Figure 1E), which is consistent with the hemolymph metabolomics data (Figure S1). Additionally, high levels of 20E titer reduced the duration of the worker bee larval feeding period and accelerated larval metamorphosis (Figure 1F).

### 3.2. Metabolic Patterns of Larvae Were Altered by Feeding with Exogenous 20E

To further investigate the regulatory effects of the 20E on the hemolymph metabolism in larvae at different developmental stages, we evaluated a central set of metabolites using a widely targeted metabolome analysis. For the metabolic profiling, hemolymph samples from both the uninfected (R) and 20E-infected (20E) larvae were collected at three development states, considered as 5-day-old (5d), 6-day-old (6d) and 7-day-old (7d), respectively (Table S2). In order to ensure the reproducibility and reliability of the data, the overlap of the mass spectrometry results of the different QC samples is shown and analyzed in Supplementary Figure S2. The results showed a high overlap of the total ion current (TIC) curves of the QC samples. Furthermore, the high correlation coefficients (|r| close to 1) of the samples in the Pearson correlation analysis of the QC samples or larval samples indicate the good stability of the performed method (Figure S3). These results confirm the stability of the overall analytical procedure and the high reproducibility and reliability of the data recorded in the present study.

**Figure 2:**
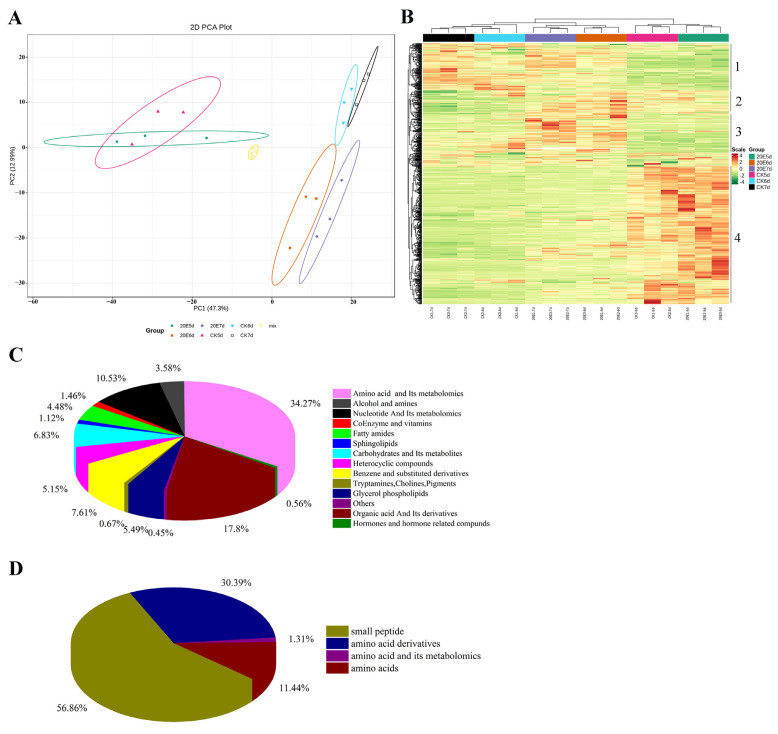
(**A**) Principal component analysis (PCA) of the different samples. Each point represents one metabolite. There were three biological repeats for each treatment. (**B**) Hierarchical clustering analysis (HCA) of widely targeted metabolomics of the different samples. Red indicates high abundance; green indicates low abundance. Metabolites were divided into four main clusters. The classification of all the detected metabolites (**C**) and amino acids (**D**) of larvae hemolymph samples.

The metabolite profiles in the samples of different larval groups, as well as the metabolite variation intra-group, were analyzed using PCA, and the results of the score plot are shown in Figure 2A. The PCA analyses showed that the first principal component (PC1) and second principal component (PC2) explained 47.3% and 12.99%, respectively, of the total variance of all samples. The second component (PC2, 12.99%) separated all the comparison groups (CK-5d vs. 20E-5d, CK-6d vs. 20E-6d, and CK-7d vs. 20E-7d), indicating remarkable differences in the metabolites of the variant larvae samples (Figure 2A). Multivariate statistical analyses were used to explore the different metabolite accumulation between samples. The HCA was used to visualize the dynamic alterations in the metabolites of 20E-injected larvae at three different developmental stages. The results of the heat-map analysis showed that the differential metabolites of larva samples were significantly different between groups, which were divided into four clusters (Figure 2B). In addition, the three biological repetitions of each larva group were clustered together, demonstrating the high reproducibility and reliability of the sample data. The PCA and heat-map analysis results indicated that 20E had a significant effect on the metabolite composition.

### 3.3. Differential Metabolites between the CK and the 20E Groups

A total number of 896 metabolites were divided into 17 classes (Table S3), including 306 amino acids and their metabolomics, 68 benzene and substituted derivatives, 32 alcohols and amines, 1 bile acid, 1 pteridine and derivatives, 13 coenzymes and vitamins, 49 glycerol phospholipids, 94 nucleotides and its metabolomics, 5 hormones and hormone-related compounds, 10 sphingolipids, 1 ester, 6 tryptamines, 61 carbohydrates and its metabolites, 159 organic acids and its derivatives, 46 heterocyclic compounds, 40 fatty amides, and 4 others (Figure 2C). Among them, amino acids and their metabolomics were the largest class of metabolites, which could be divided into four categories, including amino acids, amino acids and their metabolomics, amino acid derivatives and small peptides (Figure 2D).

**Figure 3:**
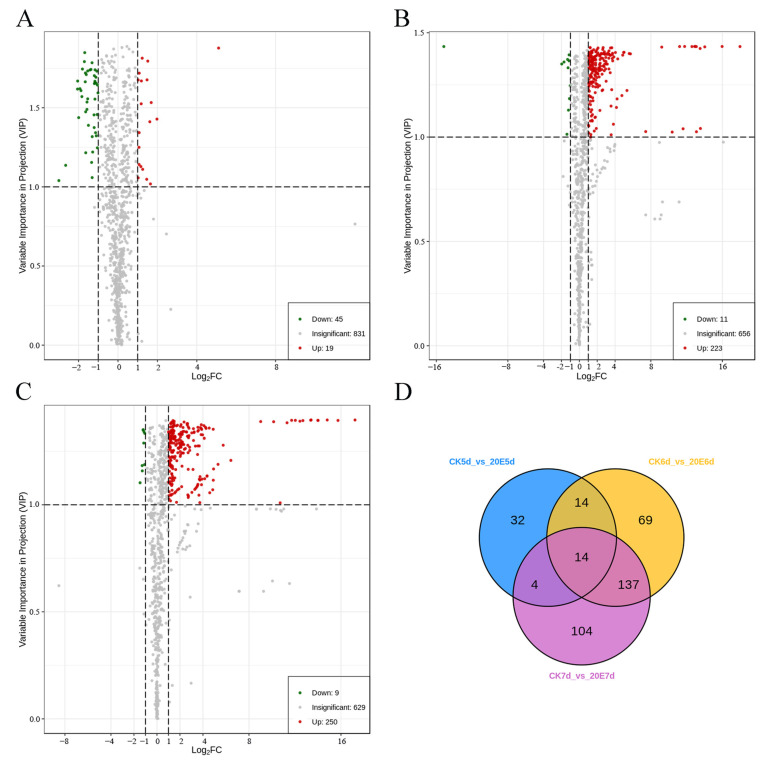
Volcano plot of the differential metabolites of (**A**) CK-5d vs. 20E-5d; (**B**) CK-6d vs. 20E-6d; (**C**) CK-7d vs. 20E-7d. (**D**) Venn diagram of the differential metabolites of CK-5d vs. 20E-5d, CK-6d vs. 20E-6d and CK-7d vs. 20E-7d. Numbers represent the identified differential metabolites of the pairwise comparisons.

OPLS-DA is a multivariate statistical analysis using supervised pattern recognition to maximize group differentiation and help screen out differential metabolites. A clear separation was found in the OPLS-DA model score plot for all the comparison groups (Figure S4), indicating significant differences in the metabolites of the variant larva samples. Differences in the metabolite expression levels between the two sample groups are represented using volcano plots (Figure 3).

Metabolites with a fold change of ≥2 or ≤0.5 and a VIP ≥ 1 were selected and regarded as significant differential metabolites. The number of upregulated metabolites was higher than the downregulated metabolites for the three paired comparisons after the 20E treatment (Figure 3A–C and Table 2). Specifically, there were significantly regulated metabolites: 64 between CK-5d and 20E-5d (19 upregulated, 45 downregulated), 234 between CK-6d and 20E-6d (223 upregulated, 11 downregulated) and 258 between CK-7d and 20E-7d (249 upregulated, 9 downregulated) (Tables S4–S6). After the 20E treatment, 6-day-old larvae exhibited more differential metabolites than the 5-day-old larvae, and 7-day-old larvae exhibited more differential metabolites than the 6-day-old larvae, indicating that 20E had a more significant effect on larval metabolites in the fasting stage than in the feeding stage. Therefore, we focused on the effects of 20E on metabolites in 6-day-old and 7-day-old larvae.

**Figure 4:**
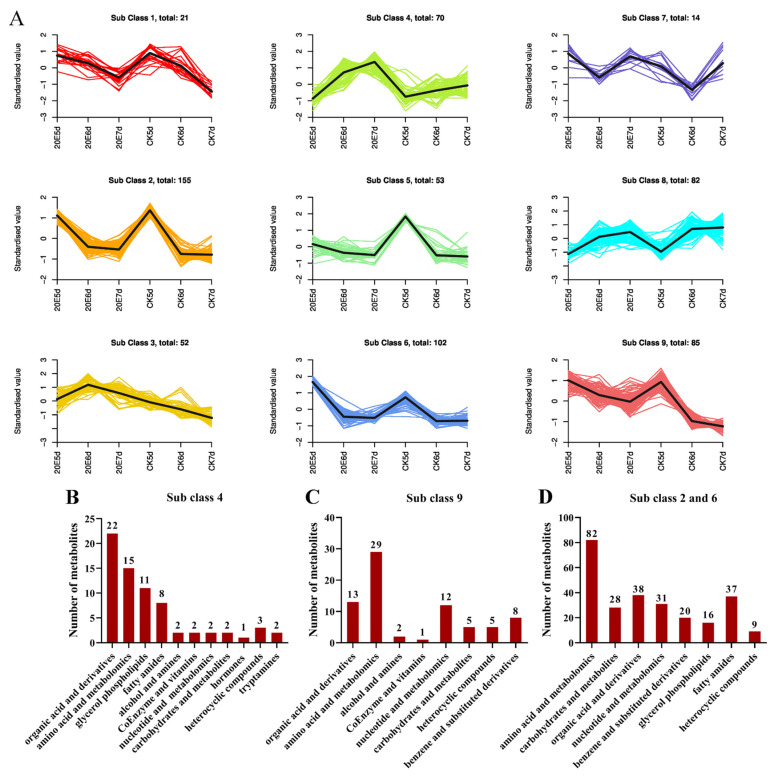
K-means clustering algorithm analysis of the dynamic alteration of the metabolites in three development states of larvae with 20E treatment. (**A**) K-means cluster was performed on Z-score normalized intensities. Classification of upregulated metabolites in Sub class 4 (**B**), Sub class 9 (**C**), Sub class 2 and 6 (**D**). Three biological replicates were analyzed for each group.

A Venn diagram was created using all these data based on the differentially expressed metabolites screened in the three paired comparisons. Common and unique metabolites were observed between the different comparison groups. Further, 151 common differential metabolites were screened in the comparison groups CK-6d versus 20E-6d and CK-7d versus 20E-7d, of which 149 were significantly upregulated, such as D-Glucose, D-(+)-sucrose, D-Melezitose and Hydroxyecdysone (Figure 3D). These differentially regulated metabolites were clustered into five classes, including amino acid and its metabolomics, carbohydrates and its metabolites, benzene and its substituted derivatives, nucleotide and its metabolomics, and organic acids and its derivatives (Table S7). Moreover, these screened differential metabolites showed six different trends among the three developmental states after the 20E treatment (Figure 4A). For example, the content of 70 metabolites increased with increasing age and was significantly higher in the 20E-fed group than in the control group (Figure 4B), whereas the content of 85 metabolites decreased with increasing age and was significantly higher in the 20E-fed group than in the control group (Figure 4C). However, the content of 257 metabolites is less varied between 6d and 7d larvae (Sub Class 2 and Sub Class 6), and most of the sugars were mainly concentrated in this trend, including D-Glucose, D-Melezitose, D-Melezitose, D-Tagatose, 3’-Sialyllactose and D-(+)-sucrose, etc. (Figure 4D).

### 3.4. KEGG Annotation and Enrichment Analysis of Differential Metabolites

**Figure 5:**
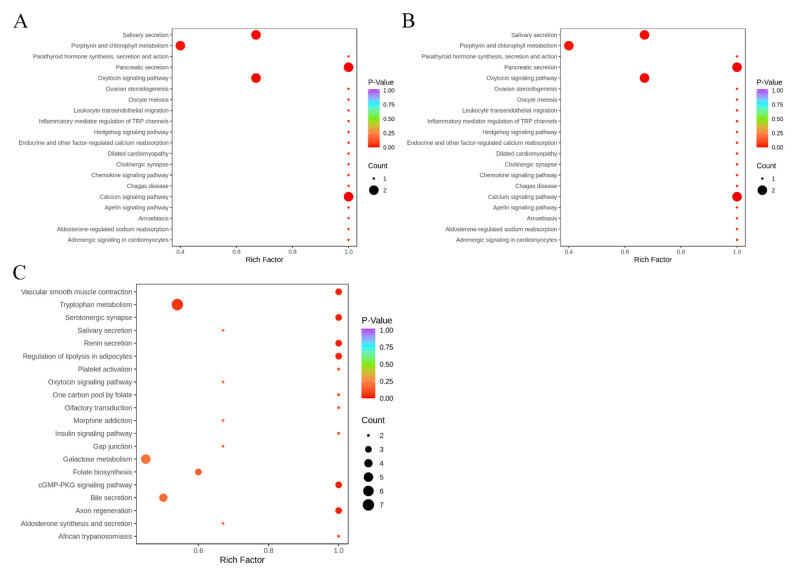
KEGG annotations and enrichment of differentially expressed metabolites of each pairwise comparison of larvae. (**A**): CK-5d vs. 20E-5d; (**B**): CK-6d vs. 20E-6d; (**C**): CK-7d vs. 20E-7d.

The screened differential metabolites were subjected to a pathway analysis by MetaboAnalyst 4.0 and a high-quality KEGG metabolic pathway database [41]. To obtain a clear understanding of all the differential metabolites for CK-5d versus 20E-5d, CK-6d versus 20E-6d and CK-7d versus 20E-7d, the overlapping metabolites were analyzed by the KEGG pathway, and the results obtained are shown in the Supplementary Material (Figure 5 and Figure S5). To further understand the metabolic changes in the different development stages of larvae in response to the 20E treatment, we subjected the differential metabolites to the KEGG pathway analysis. First, most of the differential metabolites were enriched in the metabolism. In the CK-5d versus 20E-5d group, “metabolic pathways” (ko01100) were the most represented pathways, with 72.73% of all metabolites (Figure S5A). Similar results were obtained for the other six pairwise comparisons (Figure S5B,C).

**Figure 6:**
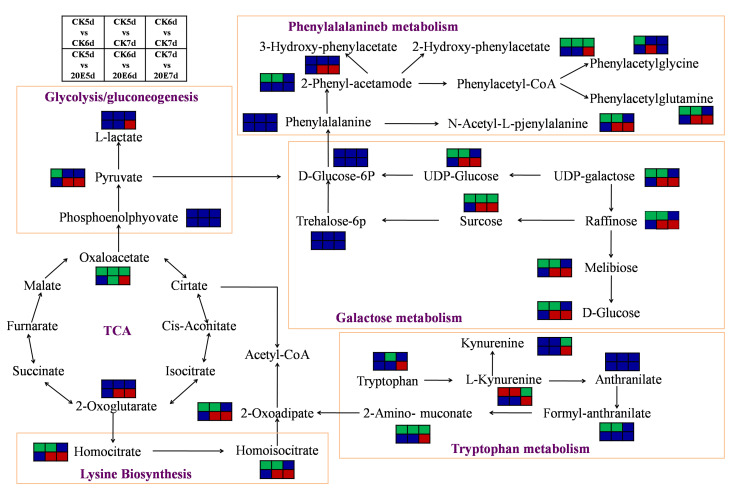
Changes in key metabolites mapped to metabolic pathways in worker bee larvae samples pairwise comparisons. Note: The small red rectangle indicates that metabolite content is significantly upregulated; the small green rectangle indicates that metabolite content is significantly downregulated; the small blue rectangle indicates no significant difference in that metabolite content.

The results of the KEGG pathway enrichment analysis showed that differential metabolites in the CK-5d versus 20E-5d, CK-6d versus 20E-6d and CK-7d versus 20E-7d groups were porphyrin and chlorophyll metabolism, glycolysis/gluconeogenesis, galactose metabolism, proline metabolism, arginine and phenylalanine metabolism, tryptophan metabolism and galactose metabolism (Figure 5, Table S8). In addition, we found that glycolysis/gluconeogenesis and galactose metabolism were significantly enriched by CK-6d versus 20E-6d and CK-7d versus 20E-7d (Figure 6), which suggests that 20E regulates the carbohydrate metabolism and maintains metabolic homeostasis in honeybee larvae.

### 3.5. 20E Promoted CM Gene Expression to Produce Glucose for Metamorphosis

**Figure 7:**
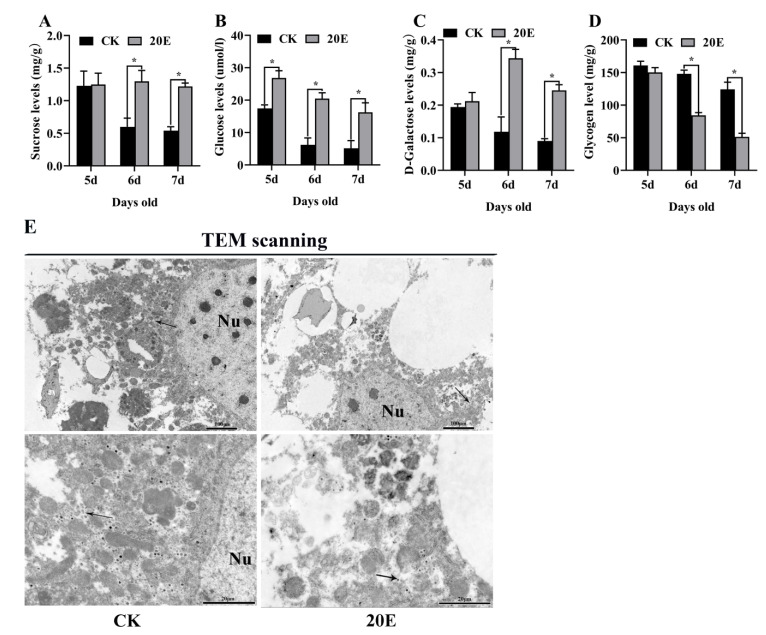
20E increased hemolymph glucose, sucrose and D-Galactose levels during metamorphosis. (**A**) The hemolymph sucrose levels during development; (**B**) the hemolymph glucose levels during development; (**C**) the hemolymph D-Galactose levels during development. Bee Ringer solution was used as the solvent control. (**D**) Glycogen levels in the fat body. (**E**) TEM observation after infection with 20E in the fat body. The arrow represented the glycogen particles. Nu: nucleus. The error bars indicate the mean ± SEM of three times repetition. The asterisks indicate significant differences from the control group (*, *p* < 0.05) by using the Student’s *t*-test.

**Figure 8:**
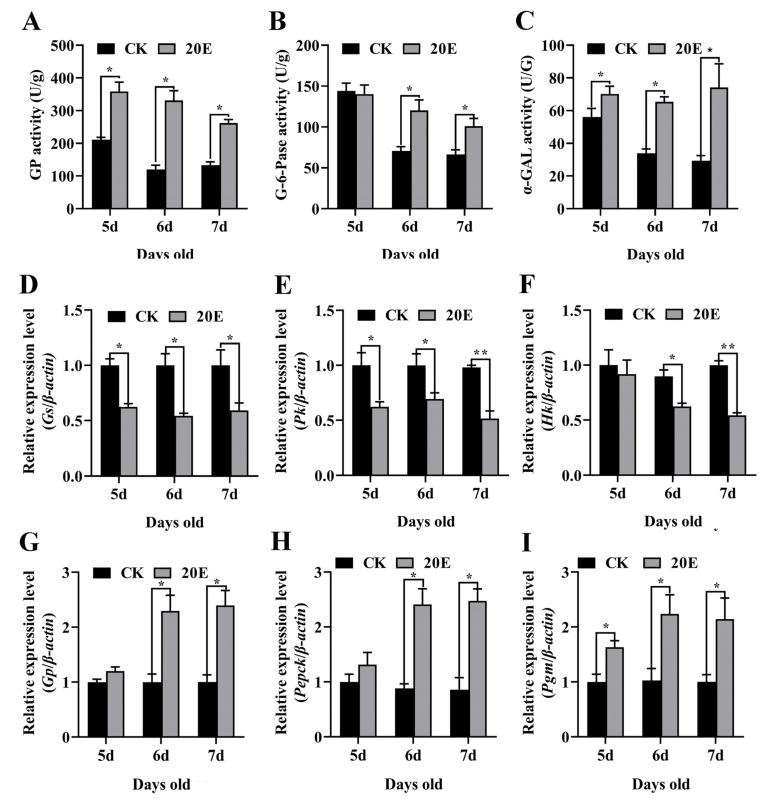
Variation in GP, G-6-Pase and α-GAL activity and related gene expression levels after 20E treatment. (**A**) The hemolymph GP activity during development; (**B**) the hemolymph G-6-Pase activity during development; (**C**) the hemolymph α-GAL activity during development. Bee Ringer solution was used as the solvent control. (**D**–**I**) qRT-PCR showing the mRNA expression levels of *Gs*, *Pk*, *Hk*, *Gp*, *Pepck* and *Pgm* after 20E treatment. The error bars indicate the mean ± SEM of three times repetition. The asterisks indicate significant differences from the control group (*, *p* < 0.05; **, *p* < 0.001) by using the Student’s *t*-test.

The widely targeted metabolomics showed that 20E increased the hemolymph glucose levels and affected CM during metamorphosis. To further verify the results, we detected the hemolymph glucose, sucrose and galactose levels at 5 d, 6 d and 7 d in larvae. We found that hemolymph glucose, sucrose and galactose levels increased significantly in 20E-fed larvae (Figure 7A–C), which is consistent with the results of the metabolomics. We also measured the glycogen levels in the fat bodies to determine the source of the high glucose levels. We found that the glycogen levels in the fat bodies dropped significantly (Figure 7D). Transmission electron microscopy (TEM) showed a decrease in the number of glycogen molecules in the fat body after the 20E treatment compared with the controls (Figure 7E), indicating that the elevated glucose levels may be caused by degradation and cessation of glycogen synthesis. To investigate this, we examined the activity of key enzymes and the expression levels of genes related to glycometabolism. The activity of the key enzymes responsible for these metabolic steps, GP, G-6-Pase and α-GAL, was higher in the 20E-fed larvae (Figure 8A–C). The mRNA encoding Glycogen synthase (Gs), Pyruvate kinase (Pk) and Hexokinase (Hk), which are key enzymes that catalyze irreversible reactions in the glycolysis pathway, were lower after the 20E treatment (Figure 8D–F). In contrast, the expression level of *Pepck*, a key enzyme encoding gluconeogenesis, was significantly higher after the 20E treatment (Figure 8H). The transcript levels of the genes encoding glycogen phosphorylase (Gp) and phosphoglucomutase (Pgm), which degrades glycogen, were increased in the worker bee larvae reared with 20E during the prepupa stage (Figure 8G–I), indicating that it promoted glycogen degradation and gluconeogenesis through upregulating the expression of related genes. These data suggested that 20E blocks glycolysis and promotes gluconeogenesis to increase the hemolymph glucose levels in honeybee larvae.

### 3.6. 20E, via EcR and USP, Regulated Gene Expression to Produce Glucose for Metamorphosis

**Figure 9:**
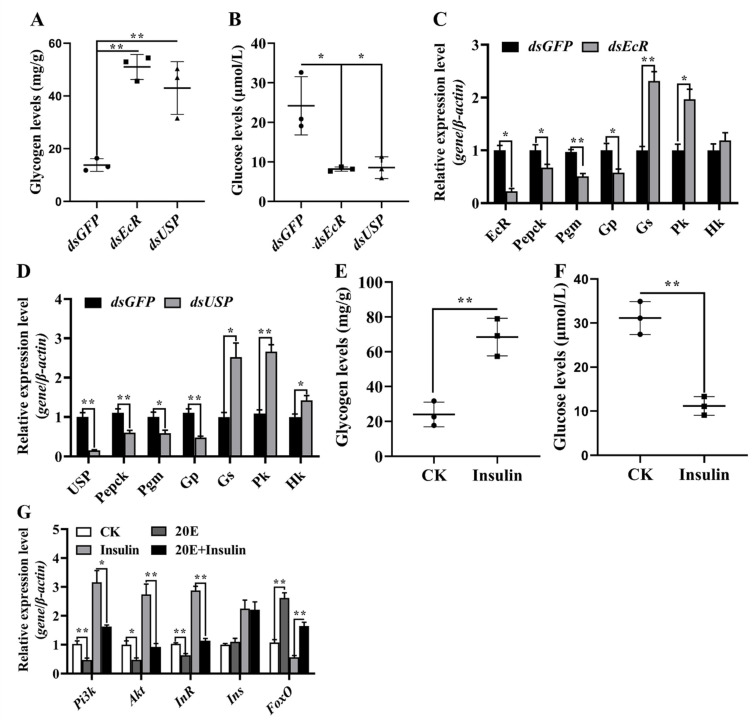
Variation in the levels of metabolites and related gene expression after *EcR* and *USP* were knocked down in larvae. (**A**,**B**) Glycogen levels in the fat body and glucose levels in the hemolymph after first injection of *dsEcR* and *dsUSP* for 48 h. (**C**,**D**) Changes in the expression levels of certain genes after *EcR* and *USP* knockdown by injection of dsRNA, as detected using qRT-PCR. (**E**,**F**) Variation in the levels of metabolites after insulin treatment. (**G**) Variation in the levels of related gene expression after 20E treatment. The error bars indicate the mean ± SEM of three times repetition. The asterisks indicate significant differences from the control group (*, *p* < 0.05; **, *p* < 0.001) by using the Student’s *t*-test.

After EcR and *USP* were knocked down in the present study, the glucose levels in the hemolymph decreased significantly, while the levels of glycogen in the fat body increased (Figure 9A,B). To determine whether 20E regulates CM gene expression through EcR and USP, we knocked down EcR and *USP* using dsRNA in 6-day-old worker bees and analyzed the CM gene transcript levels 24 h after injection. After knocking down EcR and *USP*, the mRNA levels of *Pepck* for gluconeogenesis and *Gp* for glycogen degradation were decreased, as assessed using qRT-PCR. However, the expression levels of *Hk*, *Gs* and *Pk* in glycolysis increased (Figure 9C,D). These results confirmed EcR and *USP* knockdown blocked the utilization of glycogen and stability of circulating sugar levels, thus negatively affecting metamorphosis. In addition, we found that glucose levels in the hemolymph decreased after insulin treatment. In contrast, the glycogen levels in the fat bodies increased (Figure 9E,F). 20E repressed the mRNA levels of InR, Pi3k and Akt but induced *FoxO* expression (Figure 9G). These results confirmed that 20E antagonizes insulin signaling to elevate hemolymph glucose levels.

## 4. Discussion

The study revealed that worker bee larvae reared with 20E had an altered metabolism rate and mode, and their glucose levels were significantly upregulated. Furthermore, the insect molting hormone, 20E, via the nuclear receptors, regulates glycometabolism reprogramming from glycolysis to gluconeogenesis to increase the hemolymph glucose levels.

### 4.1. Metabolome Analysis of Hemolymph Changes of Worker Bee Larvae with 20E Treatment

Steroid hormones affect various biological processes in the life cycle of insects, including metabolism, metamorphosis, reproduction and longevity. 20E plays an important role in the growth and development of honeybees. Extensive studies have shown that the endocrine signaling pathways are active in insects and transmit nutrient availability [7,8,9,10,11,12,15,42,43]. We found that feeding 20E to 2-day-old larvae until pupation resulted in smaller pupae and higher mortality compared with the controls. In addition, the 20E-fed larvae showed a lower food intake when compared to the control larvae. This suggested that 20E may lead to a reduction in nutrients in the larvae through reduced food consumption, ultimately inhibiting larval growth and pupation.

A metabolomics analysis was performed for 5-day-old larvae, 6-day-old larvae, and 7-day-old larvae fed 20E or a control solution. The PCA is an unsupervised mode of statistical analysis of multidimensional data [44]. The PCA results show the trend of metabolome separation between groups, suggesting whether there are differences in metabolome between the sample groups. Our results showed that the PCA components separated about 20E-treated larvae from solvent-treated larvae, which indicated that 20E had a significant effect on the metabolite composition in the hemolymph. Differential metabolites were identified between the 20E-fed larvae and controls. In addition, there were significantly more upregulated metabolites than downregulated metabolites for the three paired comparisons after the 20E treatment. The KEGG pathway enrichment analysis showed that the most enriched pathways between CK-5d versus 20E-5d, CK-6d versus 20E-6d and CK-7d versus 20E-7d groups were the glycolysis/gluconeogenesis, galactose metabolism, phenylalanine metabolism, tryptophan metabolism, arginine, proline metabolism and galactose metabolism. Most of the differential metabolites enriched in these metabolic pathways were significantly upregulated. In addition, the galactose metabolism was significantly enriched by comparisons of CK-6d versus 20E-6d and CK-7d versus 20E-7d.

The insect steroid hormone 20E has also been reported to regulate insect energy metabolism. In *B. mori*, it promotes the accumulation of glycogen in the fat body of fed and fasted larvae and decreases the level of trehalose in the hemolymph [8,20]. In addition, the 20E treatment also inhibited key glycolytic enzyme activities in the fat body of *Bombyx mori* larvae [22]. Ling et al. (2021) have identified a regulatory axis between the insulin-like peptide (ILPs) and 20E pathways coordinating metabolism during gonadotrophic cycles of the disease vector in *Aedis aegypti* [45]. The application of 20E allowed diapausing individuals to capacitate direct development in the presence of a significant increase in metabolism [1]. In *Helicoverpa armigera*, injection of 20E in sixth instar 6h larvae increased the glucose levels in the hemolymph compared with the DMSO controls, suggesting that 20E promotes glycogenolysis and gluconeogenesis and inhibits glucose utilization [46].

Our study showed that the levels of D-Glucose, raffinose, D-(+)-sucrose, D-Tagatose and melibiose were upregulated in the comparisons of CK-6d versus 20E-6d and CK-7d versus 20E-7d. The elevation of glucose levels in the hemolymph of honeybee larvae may be due to the inability of glucose to be absorbed into the cells and to be fully utilized during metamorphosis in the presence of 20E. The 20E hormone induces starvation through an unidentified tissue(s), such as the brain or midgut, and reduces food consumption. In the last larval stage, 20E suppresses fat body glycogen accumulation and regulates carbohydrate metabolism in *Bactrocera minax* larvae by upregulating the expression of *EcR* and *USP* [47]. In the present study, the 20E treatment caused the concentration of sugar in the hemolymph to increase, but it was under-utilized, and the nutrients in the honeybee larvae were reduced. We, therefore, propose that the differential regulation of CM, amino acid metabolism and nucleotide metabolism of 20E-fed honeybee larvae may lead to the underutilization of metabolites in the hemolymph, ultimately inhibiting bee growth.

### 4.2. The Hormone 20E, for EcR and USP, Regulates Gene Expression to Produce Glucose for Metamorphosis

Various hormones regulate glucose metabolism. In mammals, insulin promotes glucose uptake in the blood through glycolysis, increases glycogen synthesis and storage in tissue cells [48] and accelerates aerobic oxidation of glucose. In addition, ILPs play a similar role in insects through insulin/insulin-like growth factor (IGF) signaling (IIS) [49]. The hormone 20E inhibits larval feeding by competing for binding to nuclear receptors (EcR) [50] and antagonizes insulin signaling by suppressing the expression and phosphorylation of genes related to the insulin signaling pathway to increase hemolymph glucose levels [46]. In *Drosophila*, several genomic and genetic studies have shown that 20E downregulates the transcription levels of many genes involved in CM through EcR–USP at the onset of metamorphosis [18,51]. Despite a lack of measured enzymatic activity, these studies proposed that 20E–EcR–USP plays a key role in regulating metabolic reactions during pupation, guiding the change from feeding and growing larvae to immobile and non-feeding pupae [18]. In the present study, 20E blocked glycolysis and promoted gluconeogenesis by inhibiting the expression of related genes in order to block the metabolic output of glucose in the hemolymph. In addition, 20E promoted glycogen degradation and glycogen synthesis, thereby increasing the metabolic input of glucose in the hemolymph. After knocking down *EcR* and *USP*, the expression levels of *Pepck* for gluconeogenesis and *Gp* for glycogen degradation were decreased, as assessed using the qRT-PCR analysis. In contrast, the expression levels of *Hk*, *Gs* and *Pk* for glycolysis increased. Taken together, 20E regulated the programming of the glycol metabolism from glycolysis to gluconeogenesis to increase glucose during metamorphosis in honeybees via *EcR*, which, together with its heterodimeric partner *USP*, forms the functional 20E receptor. In *H. Armigera*, high glucose levels during metamorphosis are due to 20E inhibiting the expression and phosphorylation of insulin receptors [46], similar to type 2 diabetes in humans [52]. Here, we found that glucose levels in the hemolymph decreased after insulin treatment. In contrast, glycogen levels in the fat body increased. 20E repressed the mRNA levels of *InR*, *Pi3k* and *Akt* but induced *FoxO* expression. Taken together, the insect molting hormone 20E via the nuclear receptors (ECR and USP) promoted glycogen degradation and gluconeogenesis by upregulating the expression of the related genes. On the other hand, 20E antagonized insulin signaling to elevate the hemolymph glucose levels by regulating related gene expression and stopping larval growth.

## 5. Conclusions

**Figure 10:**
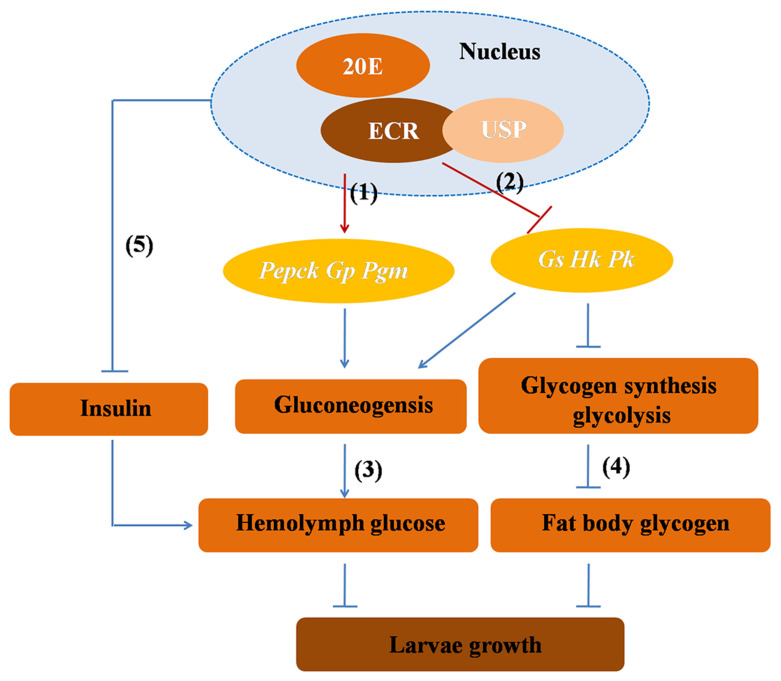
A diagram illustrating 20E regulates glycometabolism reprogramming via nuclear receptors (EcR and USP). 20E promotes the expression of *Pepck*, *Gp* and *Pgm* via nuclear receptors (1). 20E suppresses the expression of *Gs*, *Hk* and *Pk* via nuclear receptors (2). The increase in glucose after 20E treatment is due to glycogen degradation, glycolysis inhibition and gluconeogenesis (3). Glycogen levels in the fat body decreased after 20E treatment (4). 20E antagonizes IIS to elevate hemolymph glucose levels (5).

In the present study, the 20E-fed larvae demonstrated a lower food intake and the formation of smaller-sized pupae when compared to the control larvae. By using differential metabolites analysis, we found that the levels of D-Glucose were upregulated in the 20E-fed groups compared with the CK groups. In addition, we found that the elevation of the hemolymph glucose levels was the result of blocking glycolysis, enhancing the degradation of glycogen, and increasing gluconeogenesis (Figure 10). In conclusion, 20E maintained hemolymph glucose homeostasis and regulated CM through the nuclear receptor EcR-USP in honeybee larvae.

## Figures and Tables

**Table 1:** Composition of larvae diets.

Ingredients	Content (%)
Royal jelly	50.00
Glucose	6.00
Fructose	6.00
Yeast extract	1.00
Sterile water	37.00
Toal	100

**Table 2:** Classification of differential metabolites in pairwise comparisons.

Class Name	CK-5d vs. 20E-5d		CK-6d vs. 20E-6d		CK-7d vs. 20E-7d	
	Down	Up	Down	Up	Down	Up
Amino acid and its metabolomics	31	2	3	78	4	79
Benzene and substituted derivatives	1	1		19	1	22
Carbohydrates and its metabolites		1	1	16	1	17
Alcohol and amines	1			5		4
Nucleotide and its metabolomics	5	3	1	22	1	35
Hormones and hormone-related compounds		1		2		2
Tryptamines, Cholines, Pigments		1	1	1		
Glycerol phospholipids		5		7		7
Organic acid and its derivatives	4	2	3	50	1	59
Heterocyclic compounds	2		1	10		15
Coenzyme and vitamins				1		2
Fatty amides	1	3	1	12	1	7
Total	45	19	11	223	9	249

## Data Availability

Data is contained within the article or Supplementary Materials.

## Supplementary Materials

The following supporting information can be downloaded at: https://www.mdpi.com/article/10.3390/metabo13010080/s1, Figure S1. Violin plot of hydroxyecdysone. (A) CK-5d vs 20E-5d; (B) CK-6d vs 20E-6d; (C) CK-7d vs 20E-7d; Figure S2. (A) Total ions current (TIC) overlapping map of QC samples mass spectrometry results. The abscissa is the retention time of the metabolite detection. Theordinate is the ion current intensity of the ion detection (the intensity units are counts per second (cps)). (B–C) A multi-peak detection plot of the metabolites in themultiple reaction monitoring mode. (B) in positive ion mode, (C) in negative ion mode; Figure S3. The correlation analysis of QC samples or honeybee larvae samples carried out in the present method; Figure S4. The score plots of OPLS-DA pairwise comparisons of differential metabolites. (A) CK-5d vs 20E-5d; (B) CK-6d vs 20E-6d; (C) CK-7d vs 20E-7d; Figure S5. KEGG pathway annotation of the DAMs in 20E-infected larvae. (A) CK-5d vs 20E-5d; (B) CK-6d vs 20E-6d; (C) CK-7d vs 20E-7d; Figure S6. Visualization of differential regulated metabolites in CK-5d vs CK-7d (A) Pyrimidine metabolism pathway; (B) glycolysis/gluconeogenesis metabolism pathway, and CK-7d vs 20E-7d (C) Galactose metabolism pathway; Table S1. The primer sequences used for qPCR analysis and dsRNA primers sequence; Table S2. Sample information for metabolomics; Table S3. List and detailed information of the 896 metabolites identified in honeybee larvae; Table S4. List of differential metabolites between CK-5d and 20E-5d; Table S5. List of differential metabolites between CK-6d and 20E-6d; Table S6. List of differential metabolites between CK-7d and 20E-7d; Table S7. List of common differential metabolites between CK-6d vs 20E-6d and CK-7d vs 20E-7d; Table S8. Pathway analysis results from the honeybee larvae metabolomics for the six pairwise comparisons.
