# Supplementary material for: 20-Hydroxyecdysone and Receptor Interplay in the Regulation of Hemolymph Glucose Level in Honeybee (Apis mellifera) Larvae
Source: Metabolites. 2023 Jan 3;13(1):80. doi: 10.3390/metabo13010080 (PMC9865031; doi:10.3390/metabo13010080)
Supplement: Supplementary file 1 [file metabolites-13-00080-s001.zip › metabolites-2064967-supplementary-figure.pdf]

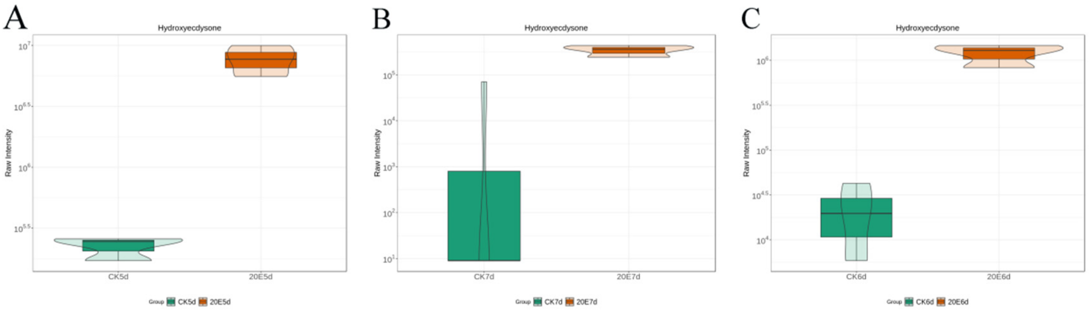

Figure S1. Violin plot of hydroxyecdysone. (A) CK-5d vs 20E-5d; (B) CK-6d vs 20E-6d; (C) CK-7d vs 20E-7d.

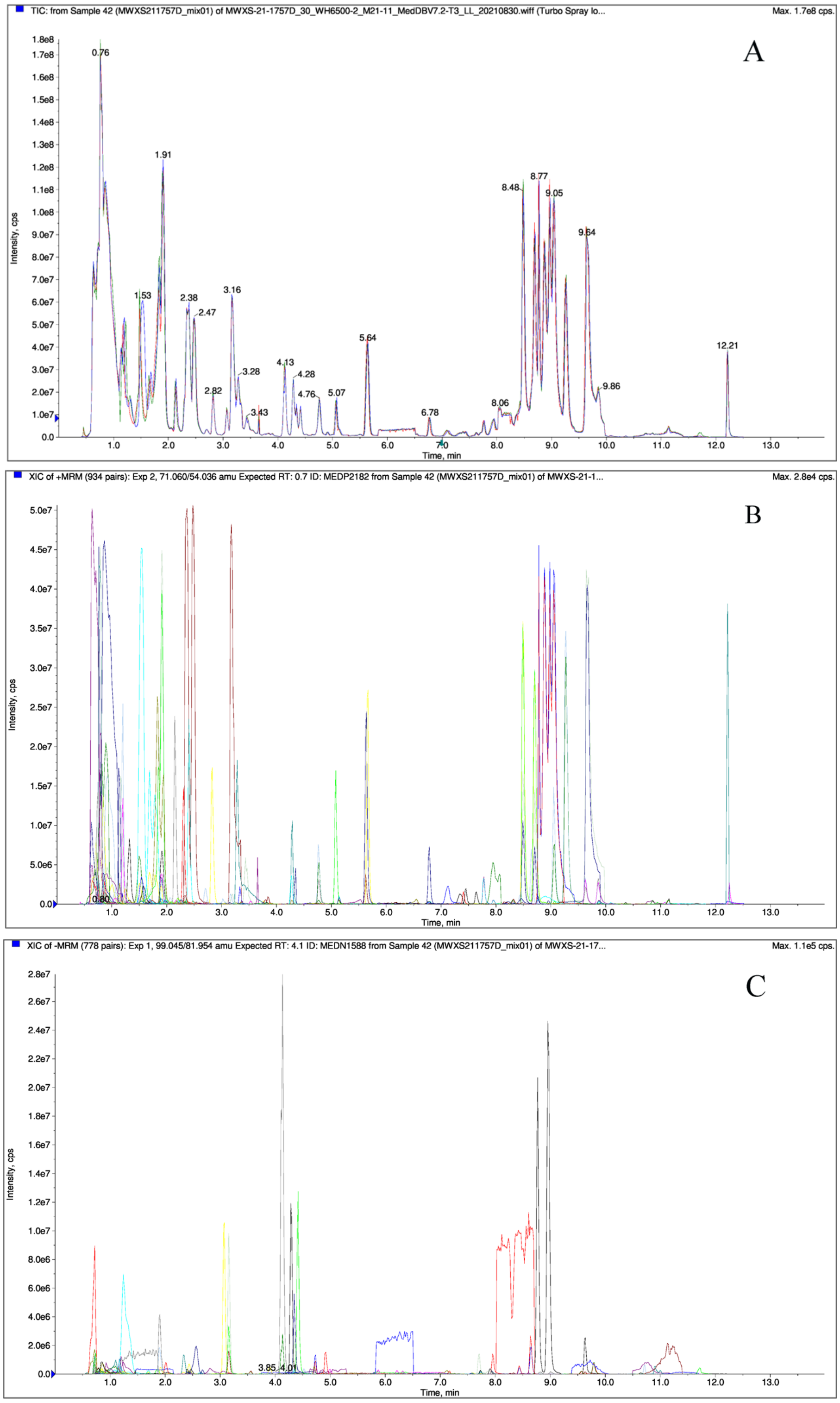

Figure S2. (A) Total ions current (TIC) overlapping map of QC samples mass spectrometry results. The abscissa is the retention time of the metabolite detection. Theordinate is the ion current intensity of the ion detection (the intensity units are counts per second (cps)). (B–C) A multi-peak detection plot of the metabolites in themultiple reaction monitoring mode. (B) in positive ion mode, (C) in negative ion mode.

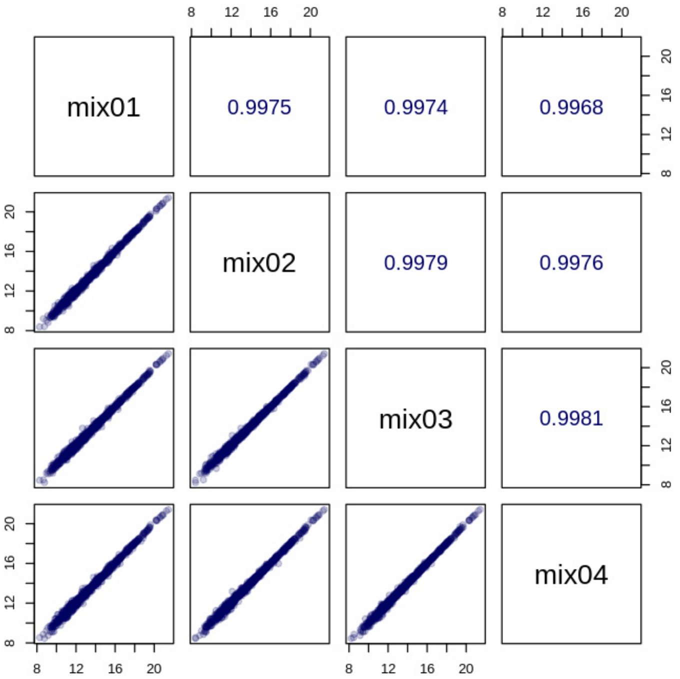

Figure S3. The correlation analysis of QC samples or honeybee larvae samples carried out in the present method.

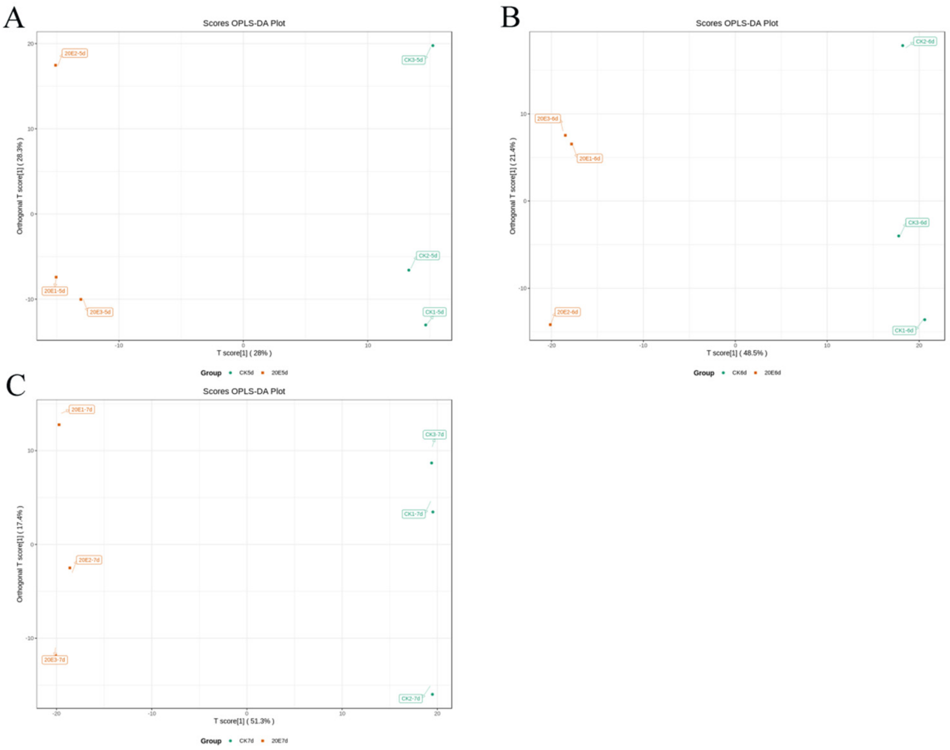

Figure S4. The score plots of OPLS-DA pairwise comparisons of differential metabolites.(A) CK-5d vs 20E-5d; (B) CK-6d vs 20E-6d; (C) CK-7d vs 20E-7d.

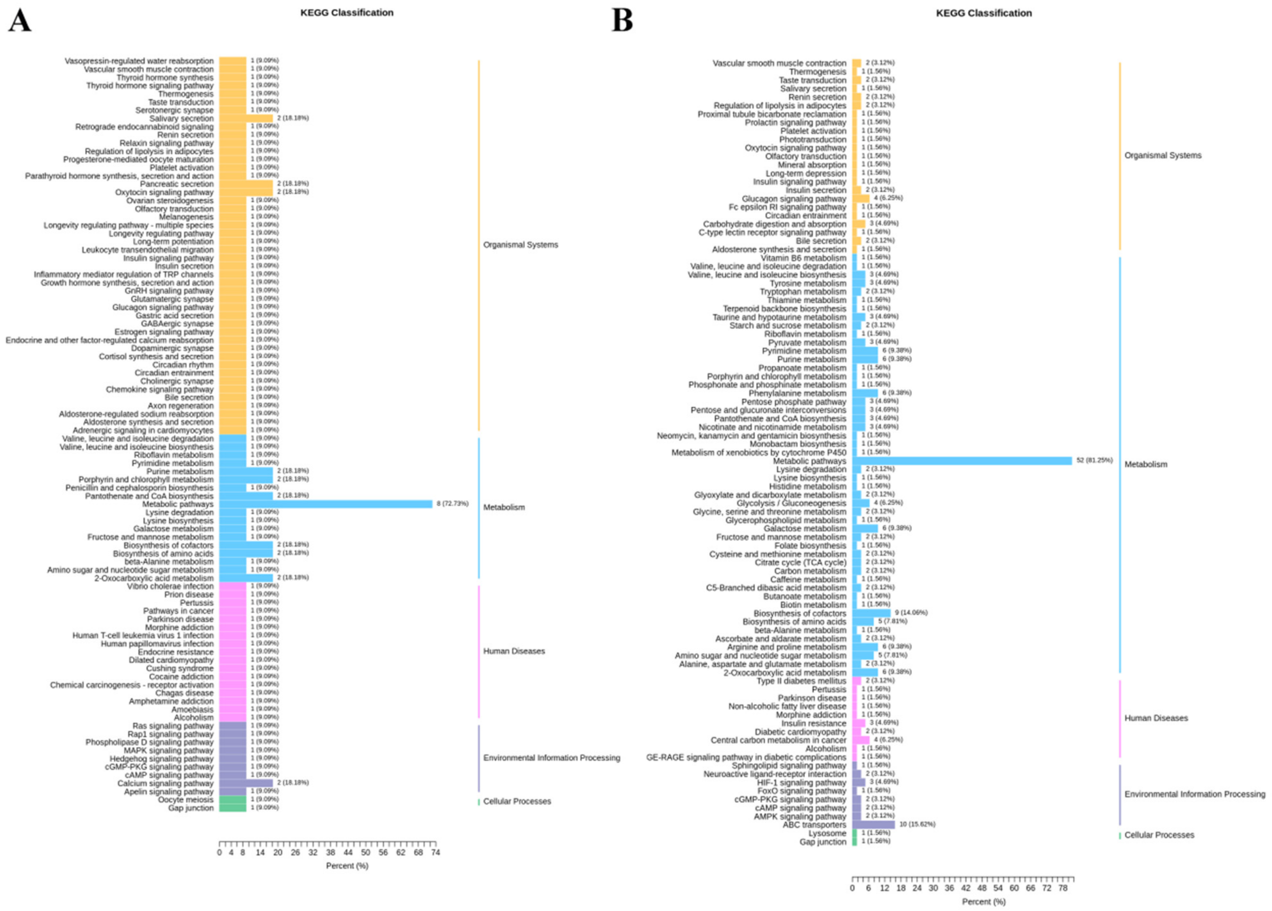
A
KEGG Classification
Vasopressin-regulated water reabsorption 1 (9.09%)
Vascular smooth muscle contraction 1 (9.09%)
Thyroid hormone synthesis 1 (9.09%)
Thyroid hormone signaling pathway 1 (9.09%)
Thermogenesis 1 (9.09%)
Taste transduction 1 (9.09%)
Serotonergic synapse 1 (9.09%)
Salivary secretion 2 (18.18%)
Retrograde endocannabinoid signaling 1 (9.09%)
Renin secretion 1 (9.09%)
Relaxin signaling pathway 1 (9.09%)
Regulation of lipolysis in adipocytes 1 (9.09%)
Progesterone-mediated oocyte maturation 1 (9.09%)
Platelet activation 1 (9.09%)
Parathyroid hormone synthesis, secretion and action 1 (9.09%)
Pancreatic secretion 2 (18.18%)
Oxytocin signaling pathway 2 (18.18%)
Ovarian steroidogenesis 1 (9.09%)
Olfactory transduction 1 (9.09%)
Melanogenesis 1 (9.09%)
Longevity regulating pathway - multiple species 1 (9.09%)
Longevity regulating pathway 1 (9.09%)
Long-term potentiation 1 (9.09%)
Leukocyte transendothelial migration 1 (9.09%)
Insulin signaling pathway 1 (9.09%)
Insulin secretion 1 (9.09%)
Inflammatory mediator regulation of TRP channels 1 (9.09%)
Growth hormone synthesis, secretion and action 1 (9.09%)
GnRH signaling pathway 1 (9.09%)
Glutamatergic synapse 1 (9.09%)
Glucagon signaling pathway 1 (9.09%)
Gastric acid secretion 1 (9.09%)
GABAergic synapse 1 (9.09%)
Estrogen signaling pathway 1 (9.09%)
Endocrine and other factor-regulated calcium reabsorption 1 (9.09%)
Dopaminergic synapse 1 (9.09%)
Cortisol synthesis and secretion 1 (9.09%)
Circadian rhythm 1 (9.09%)
Circadian entrainment 1 (9.09%)
Cholinergic synapse 1 (9.09%)
Chemokine signaling pathway 1 (9.09%)
Bile secretion 1 (9.09%)
Axon regeneration 1 (9.09%)
Aldosterone-regulated sodium reabsorption 1 (9.09%)
Aldosterone synthesis and secretion 1 (9.09%)
Adrenergic signaling in cardiomyocytes 1 (9.09%)
Valine, leucine and isoleucine degradation 1 (9.09%)
Valine, leucine and isoleucine biosynthesis 1 (9.09%)
Riboflavin metabolism 1 (9.09%)
Pyrimidine metabolism 1 (9.09%)
Purine metabolism 2 (18.18%)
Porphyrin and chlorophyll metabolism 2 (18.18%)
Penicillin and cephalosporin biosynthesis 1 (9.09%)
Pantothenate and CoA biosynthesis 2 (18.18%)
Metabolic pathways 8 (72.73%)
Lysine degradation 1 (9.09%)
Lysine biosynthesis 1 (9.09%)
Galactose metabolism 1 (9.09%)
Fructose and mannose metabolism 1 (9.09%)
Biosynthesis of cofactors 2 (18.18%)
Biosynthesis of amino acids 2 (18.18%)
beta-Alanine metabolism 1 (9.09%)
Amino sugar and nucleotide sugar metabolism 1 (9.09%)
2-Oxocarboxylic acid metabolism 2 (18.18%)
Vibrio cholerae infection 1 (9.09%)
Prion disease 1 (9.09%)
Pertussis 1 (9.09%)
Pathways in cancer 1 (9.09%)
Parkinson disease 1 (9.09%)
Morphine addiction 1 (9.09%)
Human T-cell leukemia virus 1 infection 1 (9.09%)
Human papillomavirus infection 1 (9.09%)
Endocrine resistance 1 (9.09%)
Dilated cardiomyopathy 1 (9.09%)
Cushing syndrome 1 (9.09%)
Cocaine addiction 1 (9.09%)
Chemical carcinogenesis - receptor activation 1 (9.09%)
Chagas disease 1 (9.09%)
Amphetamine addiction 1 (9.09%)
Amoebiasis 1 (9.09%)
Alcoholism 1 (9.09%)
Ras signaling pathway 1 (9.09%)
Rap1 signaling pathway 1 (9.09%)
Phospholipase D signaling pathway 1 (9.09%)
MAPK signaling pathway 1 (9.09%)
Hedgehog signaling pathway 1 (9.09%)
cGMP-PKG signaling pathway 1 (9.09%)
cAMP signaling pathway 1 (9.09%)
Calcium signaling pathway 2 (18.18%)
Apelin signaling pathway 1 (9.09%)
Oocyte meiosis 1 (9.09%)
Gap junction 1 (9.09%)
Organismal Systems
Metabolism
Human Diseases
Environmental Information Processing
Cellular Processes
0 4 8 14 20 26 32 38 44 50 56 62 68 74
Percent (%)
B
KEGG Classification
Vascular smooth muscle contraction 2 (3.12%)
Thermogenesis 1 (1.56%)
Taste transduction 2 (3.12%)
Salivary secretion 1 (1.56%)
Renin secretion 2 (3.12%)
Regulation of lipolysis in adipocytes 2 (3.12%)
Proximal tubule bicarbonate reclamation 1 (1.56%)
Prolactin signaling pathway 1 (1.56%)
Platelet activation 1 (1.56%)
Phototransduction 1 (1.56%)
Oxytocin signaling pathway 1 (1.56%)
Olfactory transduction 1 (1.56%)
Mineral absorption 1 (1.56%)
Long-term depression 1 (1.56%)
Insulin signaling pathway 1 (1.56%)
Insulin secretion 2 (3.12%)
Glucagon signaling pathway 4 (6.25%)
Fc epsilon RI signaling pathway 1 (1.56%)
Circadian entrainment 1 (1.56%)
Carbohydrate digestion and absorption 3 (4.69%)
C-type lectin receptor signaling pathway 1 (1.56%)
Bile secretion 2 (3.12%)
Aldosterone synthesis and secretion 1 (1.56%)
Vitamin B6 metabolism 1 (1.56%)
Valine, leucine and isoleucine degradation 1 (1.56%)
Valine, leucine and isoleucine biosynthesis 3 (4.69%)
Tyrosine metabolism 3 (4.69%)
Tryptophan metabolism 2 (3.12%)
Thiamine metabolism 1 (1.56%)
Terpenoid backbone biosynthesis 1 (1.56%)
Taurine and hypotaurine metabolism 3 (4.69%)
Starch and sucrose metabolism 2 (3.12%)
Riboflavin metabolism 1 (1.56%)
Pyruvate metabolism 3 (4.69%)
Pyrimidine metabolism 6 (9.38%)
Purine metabolism 6 (9.38%)
Propanoate metabolism 1 (1.56%)
Porphyrin and chlorophyll metabolism 1 (1.56%)
Phosphonate and phosphinate metabolism 1 (1.56%)
Phenylalanine metabolism 6 (9.38%)
Pentose phosphate pathway 3 (4.69%)
Pentose and glucuronate interconversions 3 (4.69%)
Pantothenate and CoA biosynthesis 3 (4.69%)
Nicotinate and nicotinamide metabolism 3 (4.69%)
Neomycin, kanamycin and gentamicin biosynthesis 1 (1.56%)
Monobactam biosynthesis 1 (1.56%)
Metabolism of xenobiotics by cytochrome P450 1 (1.56%)
Metabolic pathways 52 (81.25%)
Lysine degradation 2 (3.12%)
Lysine biosynthesis 1 (1.56%)
Histidine metabolism 1 (1.56%)
Glyoxylate and dicarboxylate metabolism 2 (3.12%)
Glycolysis / Gluconeogenesis 4 (6.25%)
Glycine, serine and threonine metabolism 2 (3.12%)
Glycerophospholipid metabolism 1 (1.56%)
Galactose metabolism 6 (9.38%)
Fructose and mannose metabolism 2 (3.12%)
Folate biosynthesis 1 (1.56%)
Cysteine and methionine metabolism 2 (3.12%)
Citrate cycle (TCA cycle) 2 (3.12%)
Carbon metabolism 2 (3.12%)
Caffeine metabolism 1 (1.56%)
C5-Branched dibasic acid metabolism 2 (3.12%)
Butanoate metabolism 1 (1.56%)
Biotin metabolism 1 (1.56%)
Biosynthesis of cofactors 9 (14.06%)
Biosynthesis of amino acids 5 (7.81%)
beta-Alanine metabolism 1 (1.56%)
Ascorbate and aldarate metabolism 2 (3.12%)
Arginine and proline metabolism 6 (9.38%)
Amino sugar and nucleotide sugar metabolism 5 (7.81%)
Alanine, aspartate and glutamate metabolism 2 (3.12%)
2-Oxocarboxylic acid metabolism 6 (9.38%)
Type II diabetes mellitus 2 (3.12%)
Pertussis 1 (1.56%)
Parkinson disease 1 (1.56%)
Non-alcoholic fatty liver disease 1 (1.56%)
Morphine addiction 1 (1.56%)
Insulin resistance 3 (4.69%)
Diabetic cardiomyopathy 2 (3.12%)
Central carbon metabolism in cancer 4 (6.25%)
Alcoholism 1 (1.56%)
GE-RAGE signaling pathway in diabetic complications 1 (1.56%)
Sphingolipid signaling pathway 1 (1.56%)
Neuroactive ligand-receptor interaction 2 (3.12%)
HIF-1 signaling pathway 3 (4.69%)
FoxO signaling pathway 1 (1.56%)
cGMP-PKG signaling pathway 2 (3.12%)
cAMP signaling pathway 2 (3.12%)
AMPK signaling pathway 2 (3.12%)
ABC transporters 10 (15.62%)
Lysosome 1 (1.56%)
Gap junction 1 (1.56%)
Organismal Systems
Metabolism
Human Diseases
Environmental Information Processing
Cellular Processes
0 6 12 18 24 30 36 42 48 54 60 66 72 78
Percent (%)

C

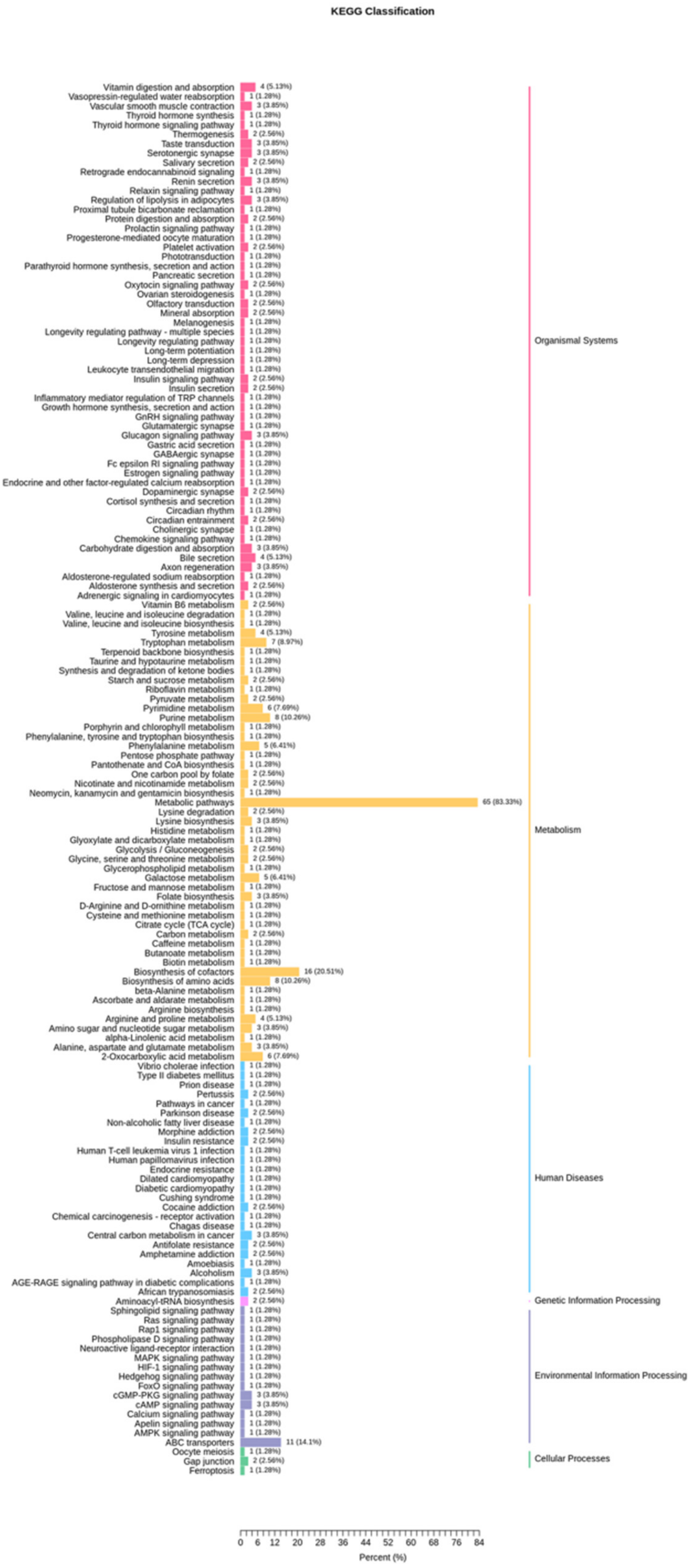

Figure S5. KEGG pathway annotation of the DAMs in 20E-infected larvae. (A) CK-5d vs 20E-5d; (B) CK-6d vs 20E-6d; (C) CK-7d vs 20E-7d.

A

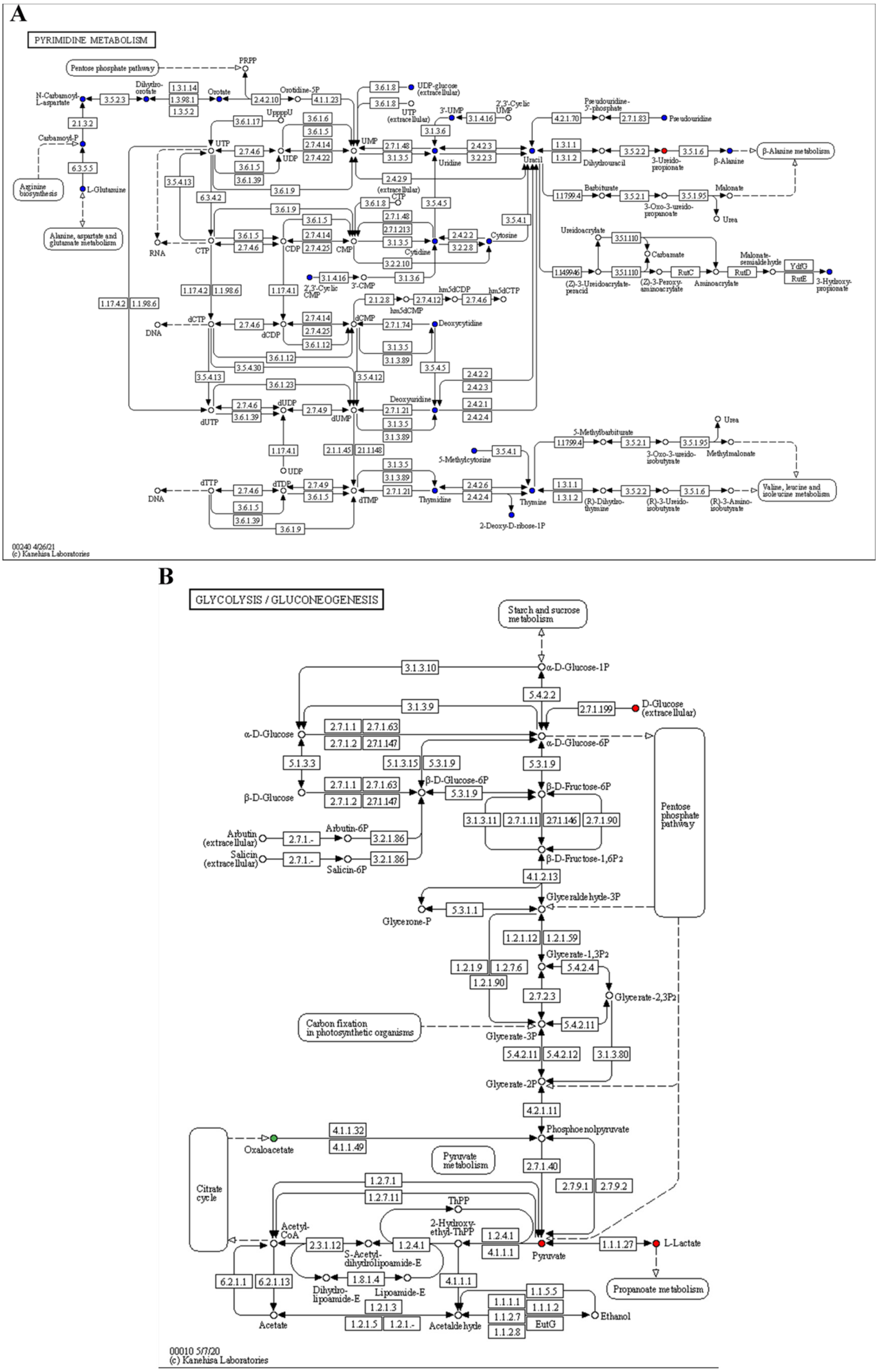
PYRIMIDINE METABOLISM
Pentose phosphate pathway
PRPP
N-Carbamoyl-L-aspartate
Dihydro-orotate
Orotate
Orotidine-5P
UDP-glucose (extracellular)
UTP (extracellular)
Carbamoyl-P
L-Glutamine
Arginine biosynthesis
Alanine, aspartate and glutamate metabolism
UTP
UDP
UMP
Uridine
Uracil
3'-UMP
2',3'-Cyclic UMP
Pseudouridine-5'-phosphate
Pseudouridine
Dihydrouracil
3-Ureido-propionate
β-Alanine
β-Alanine metabolism
Barbiturate
3-Oxo-3-ureido-propanoate
Malonate
Urea
RNA
CTP
CDP
CMP
Cytidine
Cytosine
Ureidoacrylate
Carbamate
Malonate-semialdehyde
(Z)-3-Ureidoacrylate-peracid
(Z)-3-Peroxy-aminoacrylate
Aminoacrylate
3-Hydroxy-propionate
2',3'-Cyclic CMP
3'-CMP
hm5dCDP
hm5dCTP
hm5dCMP
dCTP
dCDP
dCMP
Deoxycytidine
DNA
Deoxyuridine
dUTP
dUDP
dUMP
5-Methylcytosine
5-Methylbarbiturate
3-Oxo-3-ureido-isobutyrate
Methylmalonate
dTTP
dTDP
dTMP
Thymidine
Thymine
2-Deoxy-D-ribose-1P
(R)-Dihydro-thymine
(R)-3-Ureido-isobutyrate
(R)-3-Amino-isobutyrate
Valine, leucine and isoleucine metabolism
00240 4/26/21
(c) Kanehisa Laboratories
B
GLYCOLYSIS / GLUCONEOGENESIS
Starch and sucrose metabolism
α-D-Glucose-1P
D-Glucose (extracellular)
α-D-Glucose
α-D-Glucose-6P
β-D-Glucose
β-D-Glucose-6P
β-D-Fructose-6P
Arbutin (extracellular)
Arbutin-6P
Salicin (extracellular)
Salicin-6P
β-D-Fructose-1,6P2
Pentose phosphate pathway
Glyceraldehyde-3P
Glycerone-P
Glycerate-1,3P2
Glycerate-2,3P2
Carbon fixation in photosynthetic organisms
Glycerate-3P
Glycerate-2P
Phosphoenolpyruvate
Oxaloacetate
Citrate cycle
Pyruvate metabolism
ThPP
2-Hydroxy-ethyl-ThPP
Acetyl-CoA
S-Acetyl-dihydrolipoamide-E
Dihydro-lipoamide-E
Lipoamide-E
Pyruvate
L-Lactate
Propanoate metabolism
Acetate
Acetaldehyde
Ethanol
00010 5/7/20
(c) Kanehisa Laboratories

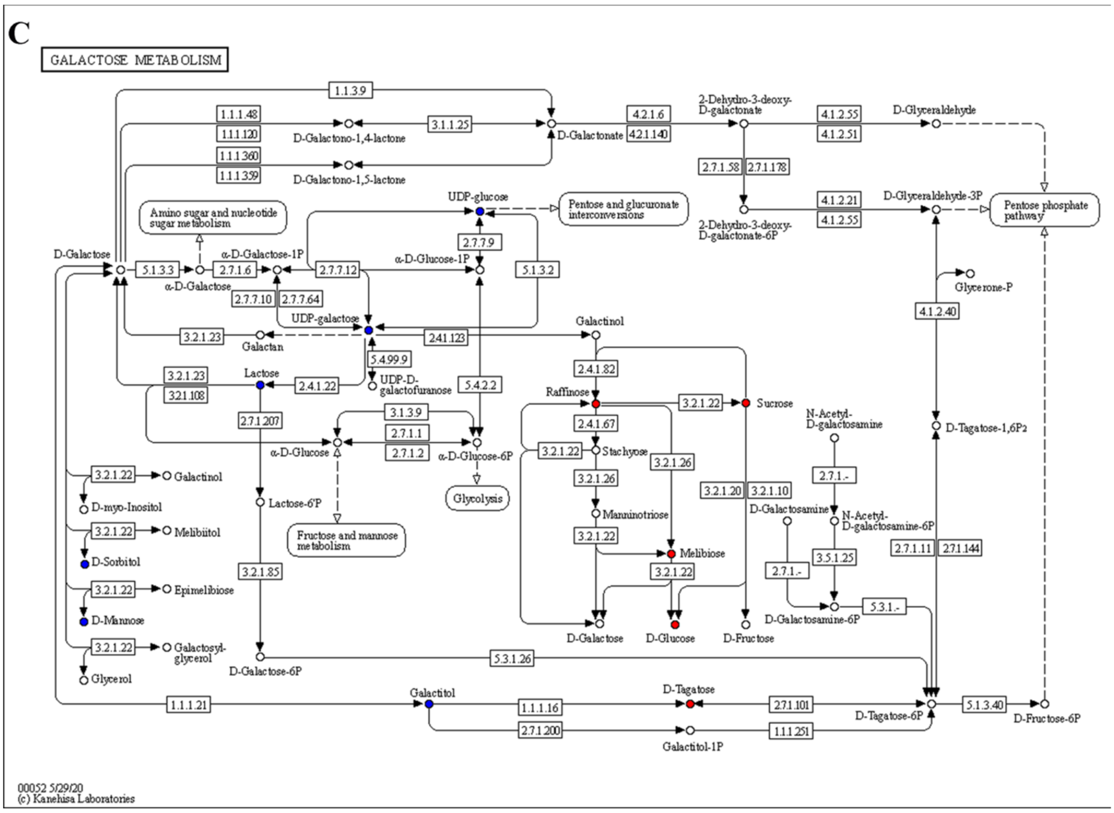

Figure S6. Visualization of differential regulated metabolites in CK-5d vs CK-7d (A) Pyrimidine metabolism pathway; (B) glycolysis/gluconeogenesis metabolism pathway, and CK-7d vs 20E-7d (C) Galactose metabolism pathway.
